# Supplementary material for: HIVprotI: an integrated web based platform for prediction and design of HIV proteins inhibitors
Source: J Cheminform. 2018 Mar 9;10:12. doi: 10.1186/s13321-018-0266-y (PMC5845081; doi:10.1186/s13321-018-0266-y)
Supplement: Supplementary file 2 — Additional file 2. Source code of HIVProtI web server. [file 13321_2018_266_MOESM2_ESM.zip › HIVprotI_Source-code/hivprotI/weka-3-6-6/doc/weka/core/Environment.html]

Environment


---


|  |  |  |
| --- | --- | --- |
| **Overview** \| **Package** \| **Class** \| **Tree** \| **Deprecated** \| **Index** \| **Help** \| **Weka's home** | |  |
| **PREV CLASS**   **NEXT CLASS** | **FRAMES**    **NO FRAMES**     **All Classes** |  |
| SUMMARY: NESTED \| FIELD \| CONSTR \| METHOD | DETAIL: FIELD \| CONSTR \| METHOD |  |


---


## weka.core Class Environment

```
java.lang.Object
  weka.core.Environment
```

**All Implemented Interfaces:**: RevisionHandler

---

``` public class Environment extends java.lang.Object implements RevisionHandler ```

This class encapsulates a map of all environment and java system properties.
There are methods for adding and removing variables as well as a method for
replacing key names (enclosed by ${}) with their associated value in Strings.

**Version:**
:   $Revision: 5562 $

**Author:**
:   Mark Hall (mhall{[at]}pentaho{[dot]}com)

---

**Constructor Summary**

`Environment()`


| **Method Summary** | |
| --- | --- |
| `void` | `addVariable(java.lang.String key, java.lang.String value)`             Add a variable to the internal map. |
| `static boolean` | `containsEnvVariables(java.lang.String source)`             Tests for the presence of environment variables. |
| `java.lang.String` | `getRevision()`             Returns the revision string. |
| `static Environment` | `getSystemWide()`             Get the singleton system-wide (visible to every class in the running VM) set of environment variables. |
| `java.util.Set<java.lang.String>` | `getVariableNames()`             Get the names of the variables (keys) stored in the internal map. |
| `java.lang.String` | `getVariableValue(java.lang.String key)`             Get the value for a particular variable. |
| `static void` | `main(java.lang.String[] args)`             Main method for testing this class. |
| `void` | `removeVariable(java.lang.String key)`             Remove a named variable from the map. |
| `java.lang.String` | `substitute(java.lang.String source)`             Substitute a variable names for their values in the given string. |

**Methods inherited from class java.lang.Object**

`equals, getClass, hashCode, notify, notifyAll, toString, wait, wait, wait`

**Constructor Detail**

### Environment

```
public Environment()
```


**Method Detail**

### getSystemWide

```
public static Environment getSystemWide()
```

:   Get the singleton system-wide (visible to every
    class in the running VM) set of environment
    variables.

    :   **Returns:**: the system-wide set of environment variables.

---


### containsEnvVariables

```
public static boolean containsEnvVariables(java.lang.String source)
```

:   Tests for the presence of environment variables.

    :   **Parameters:**: `source` - the string to test **Returns:**: true if the argument contains one or more environment variables

---


### substitute

```
public java.lang.String substitute(java.lang.String source)
                            throws java.lang.Exception
```

:   Substitute a variable names for their values in the given string.

    :   **Parameters:**: `source` - the source string to replace variables in **Returns:**: a String with all variable names replaced with their values **Throws:**: `java.lang.Exception` - if an unknown variable name is encountered

---


### addVariable

```
public void addVariable(java.lang.String key,
                        java.lang.String value)
```

:   Add a variable to the internal map.

    :   **Parameters:**: `key` - the name of the variable: `value` - its value

---


### removeVariable

```
public void removeVariable(java.lang.String key)
```

:   Remove a named variable from the map.

    :   **Parameters:**: `key` - the name of the varaible to remove.

---


### getVariableNames

```
public java.util.Set<java.lang.String> getVariableNames()
```

:   Get the names of the variables (keys) stored in the
    internal map.

    :   **Returns:**: a Set of variable names (keys)

---


### getVariableValue

```
public java.lang.String getVariableValue(java.lang.String key)
```

:   Get the value for a particular variable.

    :   **Parameters:**: `key` - the name of the variable to get **Returns:**: the associated value or null if this variable is not in the internal map

---


### main

```
public static void main(java.lang.String[] args)
```

:   Main method for testing this class.

    :   **Parameters:**: `args` - a list of strings to replace variables in (e.g. "\${os.name} "\${java.version}")

---


### getRevision

```
public java.lang.String getRevision()
```

:   Returns the revision string.

    :   **Specified by:**: `getRevision` in interface `RevisionHandler`
    :   **Returns:**: the revision


---


|  |  |  |
| --- | --- | --- |
| **Overview** \| **Package** \| **Class** \| **Tree** \| **Deprecated** \| **Index** \| **Help** \| **Weka's home** | |  |
| **PREV CLASS**   **NEXT CLASS** | **FRAMES**    **NO FRAMES**     **All Classes** |  |
| SUMMARY: NESTED \| FIELD \| CONSTR \| METHOD | DETAIL: FIELD \| CONSTR \| METHOD |  |


---
